# Pilot Study of a Group-Based Psychosocial Trauma Recovery Program in Secure Accommodation in Scotland

**DOI:** 10.1007/s10896-017-9921-8

**Published:** 2017-06-11

**Authors:** Ian Barron, David Mitchell, William Yule

**Affiliations:** 1 Old Medical School, School of Education and Social Work, University of Dundee, Dundee, 0113 UK; 2 Rossie Young Peoples Trust, Montrose, UK; 3 Kings College London, University of London, London, UK

**Keywords:** Secure care, Trauma recovery, Program fidelity, Trauma-informed

## Abstract

The current study is the first to implement and evaluate a group-based trauma-specific program for adolescents in a secure accommodation facility in Scotland. A randomized control and qualitative pilot study compared an intervention group (*n* = 10), who received Teaching Recovery Techniques, to a waitlist control group (*n* = 7). Measures included subjective units of disturbance (SUDs), standardized trauma symptom questionnaires, and analysis of behavior monitoring logs. Adolescent interviews (*n* = 10) and a presenter focus group (*n* = 4) assessed program experience and views on future development. Sessions were videoed and analyzed for program adherence. Analysis involved MANOVA, and a quasi-qualitative thematic approach for participant views. Adolescents reported high SUDs and a range of trauma symptoms. A large effect size was found for reduced SUDs (*d* = 1.10) and positive trends were identified for symptoms and behavior change in the intervention group. Program adaptations included smaller groups, the use of visual materials and liaison with care staff to facilitate generalization. Recommendations are made for program development and large scale evaluation.

The current study is the first to examine the implementation and evaluation of introducing a trauma-specific intervention into a Scottish secure facility. To assess the impact of a trauma-specific program on reducing adolescent trauma symptoms the study utilized a randomized control design with standardized trauma symptom measures. Analysis of behavior monitoring records identified any reductions in behavior incidents and qualitative interviews explored participant experiences in order to discover adaptations needed for future implementation. The study built on an exploratory study of traumatization in adolescents in a secure facility in Scotland (Barron and Mitchell 2016).

Due to the limited literature in Scotland, studies in the United States of America (USA) have been used to set the wider context for the current study. The extant literature in the USA has found high rates of trauma exposure among adolescents in secure facilities. Dierkhising et al. (2013) found rates as high as high as 90 %, three times higher than in the general population. A recent large scale study (*n* = 64,329) of Adverse Childhood Experiences (ACE) of juvenile offenders, building on the earlier 2013 diverse urban community study of 1784 adults, found 10 different adversities: emotional and physical neglect; family violence and separation; mental health problems; imprisonment of a relative; emotional, physical, and sexual abuse; and substance misuse (Baglivio et al. 2014). Ford et al. (2012) have also found traumatic loss and community violence prevalent in this population and adolescents while in secure facilities experience peer violence and staff restraint (Sickmund and Puzzanchera 2014). Girls, compared to boys in secure facilities are two to four times more likely to report sexual abuse and sexual assault (Dierkhising et al. 2013). Because of the growing evidence of a gender difference in the extent of traumatization for girls, Kerig and Ford (2014) argue that programs in secure facilities need to be responsive to gender.

In comparison, the only Scottish study to explore traumatization rates in a secure facility found adolescents who participated in a trauma history interview (*n* = 17) reported *m* = 8.47 traumatic events on average, ranging from four to twelve events. Similar to the ACE study, sixteen different types of event were identified. All adolescents reported sudden traumatic losses, physical assault and frequent placement changes. Nearly 90% reported physical abuse by relatives (*n* = 15) and over 70% (*n* = 12) experienced domestic violence and sexual abuse. The latter included all 11 girls in the sample. Neglect was reported by 59% (*n* = 10). Finally, hospitalization (*n* = 9) and emotional abuse (*n* = 7) was experienced by 53% and 41% of adolescents, respectively (Barron and Mitchell 2016). In summary, it appears traumatic events are pervasive in the lives of adolescents in secure facilities in Scotland as well as in the USA. Despite the extent of adolescent traumatization, over the past few decades, secure facilities in Scotland and USA have delivered behavior programs in an attempt to reduce delinquent behavior. The little evidence that exists, suggests poor outcomes, high recidivism rates and a lack of long-term behavior change (Trulson et al. 2005). Mears et al. (2011), argue that this because the underlying trauma that drives the behavior is not being addressed. Van der Kolk et al. (2009) conceptualized this underlying trauma as developmental trauma disorder. Symptoms include pervasive and include posttraumatic stress, dissociation, difficulties with trust, negative self-concept, emotional dysregulation, behavioral difficulties, lack of empathy, and a foreshortened view of the future. As a consequence, there has been an increasing shift to providing trauma-informed milieu and the beginnings of delivering trauma-specific programs in secure facilities in the USA (Ford and Blaustein 2013).

Recent studies in secure facilities have confirmed the link between a history of abuse and trauma to the symptoms of posttraumatic stress, anxiety, depression, and suicide (Bhatta et al. 2014; Dierkhising et al. 2013). In Scotland, Barron and Mitchell (2016) found adolescents reported high levels of posttraumatic stress (65%), depression (65%), and to a lesser extent dissociation (18%). Not surprisingly, given the levels of abuse and trauma experienced, the extent of trauma symptoms for adolescents in secure facilities have been found to be considerably higher than in the general population (Stimmel et al. 2014). Despite these mental health concerns, placement in a secure facility in Scotland occurs primarily because of delinquent behavior that is difficult for child care services to manage, and poses a risk to self and others. Self-harm, attempted suicides, hospitalizations, vandalism, theft, fire-raising, substance misuse, prostitution, and absconding from homes and care placements are all recorded in secure facility case files. Specific references to trauma and trauma symptoms, however, are characterized by their absence (Barron and Mitchell 2016). Although considerable research establishes the link between child abuse, trauma symptoms, and delinquent behavior for adolescents (Olafson et al. 2016) programs for addressing trauma symptoms for youth in secure facilities are in their infancy (Kerig 2013; Ford et al. 2013). This may be partly due to under-reporting of adolescent traumatization as well as poor recognition of trauma symptoms by professionals in the child care field (Finkelhor et al. 2012; Barron and Mitchell 2016). As a consequence of the limited recognition of trauma, evaluation of trauma-specific programs and trauma-sensitive milieu is sparse. The Trauma and Affect Regulation (TARGET) is one of the few programs where a series of studies have been conducted (Ford and Hawke 2012; Ford et al. 2012; Marrow et al. 2012). Marrow et al. (2012) evaluated the Trauma and Affect Regulation (TARGET) milieu program in a non-randomized study of 38 adolescents with mental health difficulties in different units. The study found significant reductions in depression, threatening behavior and restraint as well as increases in hopefulness compared to a treatment as usual group. Ford and Hawke (2012) evaluating the same program discovered that intervention within the first week of placement, led to a 54% reduction in behavior incidents and a significant reduction in recidivism following program delivery. There is also evidence for the effectiveness of the trauma-informed group milieu Sanctuary model (Rivard et al. 2005). The Sanctuary model, with its focus on developing safety, the management of emotion and ways of dealing with loss, increased the capacity for institutions to operate as a therapeutic community (Rivard et al. 2003). The Structured Psychotherapy for Adolescents Responding to Chronic Stress (SPARCS: Habib et al. 2013), a trauma-specific program, aims to develop adolescent self-regulation; self-concept, relationships, meaning-making and future hope. Within a small scale pilot study (*n* = 24) adolescents gains in anxiety, depression, physical complaints and behavior. More recently Olafson et al. (2016) evaluated the trauma-focused group treatment Trauma and Grief Component Therapy for Adolescents (*n* = 77) alongside trauma-informed whole staff training in Think Trauma. The authors concluded that it was possible to deliver a trauma-specific program in juvenile justice resulting in a significant reduction in PTSD, depression and anger. As Ford and Blaustein (2013) argue, it appears group-based trauma-specific approaches have the potential to influence the nature of the secure environment for adolescents with complex and challenging needs leading to improved behavior and mental health outcomes.

In Scotland, trauma-specific approaches have yet to be evaluated in secure facilities (Barron and Mitchell 2016). The current study is a pilot study that introduces and evaluates a trauma-specific program into a secure facility in Scotland. The Children and War Foundation’s Teaching Recovery Techniques (TRT: Smith et al. 2008) was selected because of its growing evidence for use with adolescent populations who have experienced cumulative trauma and display PTSD and other trauma symptoms (Barron et al. 2013, 2016). TRT focuses on teaching adolescents coping skills to deal with the symptoms of posttraumatic stress. Both TRT and SPARCS are based on cognitive behavioral therapy, however, TRT is half the number of sessions. This was important because of the short duration placements of less than three months in Scotland. The uniqueness of this pilot study is in its application of transferring a trauma-specific recovery program for adolescents who experienced cumulative war and domestic violence to a population who have experienced domestic trauma and are placed in a secure facility.

## Methods

### Research Design

This was the first study in Scotland to introduce and evaluate a trauma-specific program into a secure facility in Scotland. As this was a novel context for the implementation of TRT, a pilot study was conducted. A randomized control design was utilized in order to compare adolescents who received TRT with those on a waitlist. Trauma history interviews with subjective units of disturbance scores were used to assess adolescent trauma exposure and related subjective disturbance before and after intervention. As a normative comparison, standardized measures of trauma symptoms were administered. Staff completed a standardized measure to compare with adolescent responses. Interviews were held with adolescents and a focus group was conducted with presenters to explore and compare their experience of the program and to gain ideas for future program development. Program sessions were videoed and a small sample analyzed to assess program fidelity and adaptation in a new context. Interview and focus group data were analyzed using a quasi-qualitative approach in order to identify the nature and frequency of participant views. Ethics approval was granted from a University Research Ethics Committee. Active signed informed consent was required for parents, adolescents and staff. Participants could withdraw at any time.

### Sample

**Fig. 1:**
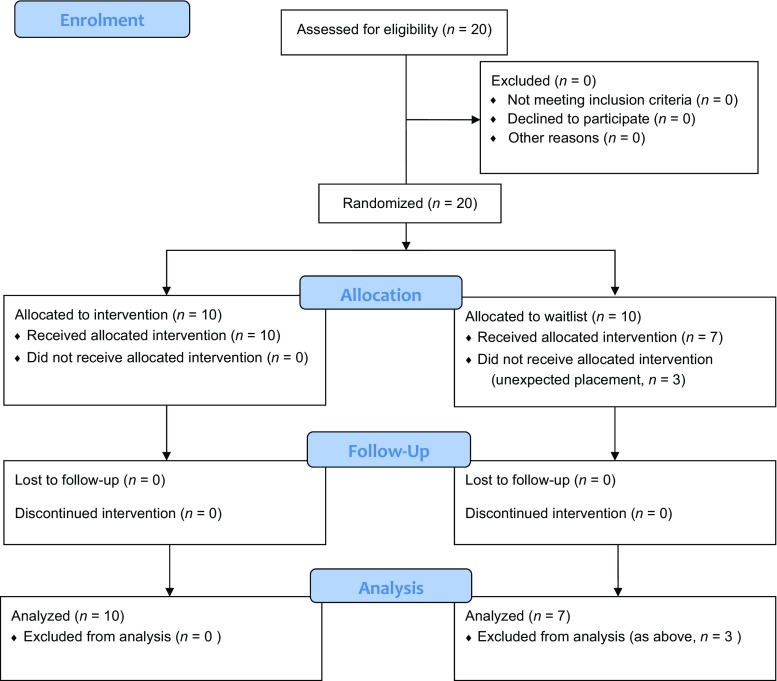
Participant flow diagram

The study was conducted in one of five secure facilities in Scotland. The facility was located outside a small Scottish town in a rural location. The maximum number of adolescents in the facility was 20. Adolescents were placed from all over Scotland. To date, the facility had focused on delivering behavior change programs. Twenty adolescents, the corpus sample, from four different care units, were selected and then randomly allocated to intervention and waitlist control conditions (See Fig. 1) using the toss of a coin. Three male adolescents from the waitlist were moved out of the facility by their local authority for financial reasons and were not able to participate in the research. This resulted in 10 adolescents in the intervention and seven in the usual social education, no-treatment waitlist. Waitlist adolescents received TRT a month following post-test. Adolescents were aged 14–18 years (*m* = 15.05 years; *SD* = 1.12) in intervention and waitlist. The study sample consisted of 11 females and 6 males (7 females and 3 males) received the intervention and 4 females and 3 males were on the waitlist. All were Scottish and Caucasian, from unemployed families, from a wide geographical spread across Scotland. All three presenters were experienced program workers. Two were social workers and one had a psychology background. All were trained to deliver a variety of mental health and behavior change programs. Adolescents were in secure from 1 to 7 months prior to TRT with an average period of 4 months. Nine of the adolescents had only been in placement for two months.

### The Program

TRT is a trauma-specific program based on cognitive behavioral theory that focuses on normalizing the trauma response; teaching strategies for intrusive memories, hyper-arousal, and avoidance symptoms of PTSD as well as coping with loss. TRT was originally developed for adolescents who experienced disaster situations, such as earthquakes, and war trauma. Content includes: (i) case studies as exemplars for psychoeducation on traumatic events, normalizing resultant symptoms, and stimulating the sharing of traumatic events; (ii) relaxation techniques and positive cognitions to help with emotional dysregulation; (iii) brief exposure for trauma reminders; and (iv) systematic desensitization of anxiety and anger hierarchies for avoidance. Because of short concentration spans and social skill difficulties, adolescents received an adapted version of TRT. Sessions were shorter, 40 min on average, and delivered twice weekly over seven weeks, rather than weekly two hour sessions. Two program workers were present during delivery, one to present, the other to support. Presenters received a-three day training by an expert trainer (Bill Yule, Emeritus Professor King’s College London) from the Children and War Foundation covering program values, content and processes. Training methods mirrored program activities and included information giving, modeling, experiential learning, reflection, and feedback. TRT was delivered to the intervention group during school time and over three phases (May, October and February) with four, four and two adolescents in the intervention groups respectively. Presenters received group supervision by the principle researcher, following each phase of delivery. This involved affirming adherence to TRT protocols, making adaptations within theoretical guidance and being responsive to adolescents.

### Program Fidelity

All sessions were video recorded for fidelity analysis. The fidelity framework, based on the structure of sessions and the scripted activities in the TRT manual, was established prior to delivery. Analysis of two sessions by the principle researcher (Reader in Trauma Studies) and co-researcher (team manager) led to adaptation of the fidelity framework to include the nature and extent of protocol adaptation, and presenter non-verbal and verbal communication. Four sessions were analyzed with the adapted framework. Duration of communicative behavior is reported as a percentage of session length. Inter-rater reliability, involved analysis of the four sessions and is reported as Cohen’s kappa *k.*

### Trauma History Interview

Greenwald’s (2005) protocol was used to enable adolescents to list and rate traumatic events. This involved asking adolescents at what age salient events occurred in their lives, what events occurred, and how disturbing these events are currently on a scale from 0 to 10, where 0 is no disturbance, and 10 is the most distressing things can be, i.e., a subjective units of disturbance scale (SUDs). Adolescents in TRT and waitlist were interviewed 2 weeks prior to and following TRT by the researcher with a program worker present. Interviews took 30 min on average.

### Standardized Measures

#### Posttraumatic Stress

A battery of six standardized measures was administered. The Impact of Events Scale (CRIES-13; Smith et al. 2003) assesses the likelihood of PTSD (intrusion, avoidance and arousal) on a 13 item 4 point scale, with a clinical cut off of >17. CRIES-13 shows good internal consistency (Cronbach alpha coefficient .80).

#### Depression

The Moods and Feelings Questionnaire (MFQ: Angold et al. 1995) for 7–18 year olds assesses the likelihood of depression and shows moderate to high criterion validity of .86. Thirteen items are measured on a 3 point scale. The clinical cut off is 11.

#### Traumatic Grief

The Traumatic Grief Inventory for Children (TGIC: Dyregrov et al. 2001) has 24 questions over a 5 point scale and assesses the symptoms of traumatic grief such as sadness, longing, loneliness and intrusive thoughts. Currently there is no clinical cut-off and as such is an experimental measure.

#### Dissociation

The Adolescents Dissociative Experiences Scale (ADES: Armstrong et al. 1997) assesses the levels of dissociation over thirty items on a 0–10 scale for 10 to 21 year olds. A score of 3.7 or over would be clinically concerning and suggestive of dissociation. The ADES-A has high internal consistency with a Cronbach alpha of .94 (Farrington et al. 2001).

#### Mental Health

The Strengths and Difficulties Questionnaire (SDQ: Goodman 1997) for 11–17 year olds assesses adolescents’ wider mental health difficulties including positive and negative attributes on 25 items over five subscales of five items each, over a three point scale. Cronbach’s alpha is .64. Facility staff who were most familiar with adolescents completed the SDQ-T in order to compare participant ratings with staff ratings, Cronbach alpha .70. (Muris et al. 2003). Questionnaires were administered to participants by their program workers 2 weeks prior to and after TRT delivery.

### Behavior Monitoring

Behavior monitoring involved the analysis of 24 h daily observation sheets. These were completed by care workers in the units for each participant. The frequency of recorded ‘aggressive behaviors towards self or others’ and participant ‘upset’ was calculated and analyzed pre and post-test for TRT and waitlist. Aggression was defined as any behavior involving physical, verbal or non-verbal threat or actions towards self or another, e.g. self-harm, hitting, shouting in threatening manner, and standing over in menacing way. Upset was defined as any recorded comment that described a negative shift in emotion, e.g. upset, distressed, and angry.

### Adolescent Interviews

Interviews were held with adolescents 1 month post TRT to assess their subjective experience of the program. Adolescents (*n* = 10) were asked what they thought of TRT including: whether it was helpful and in what ways; which parts worked best; what was learned; what strategies were applied in real life; how likely is it that they will use the strategies in real life (on a zero to ten scale); if any negative consequences were experienced and what would improve TRT? Adolescent responses were recorded verbatim by the researcher and checked for accuracy by the program worker at the time of interview. Analysis involved a quasi-qualitative thematic analysis that utilizes identification of meaningful codes and themes from statements as well as the frequency counts of statements under each code. A quasi-qualitative analysis was chosen in order to not only identify participant meanings but also to get a measure of how often the meanings were reported by adolescents and potential measure of importance. The steps within the quasi-qualitative analysis were: familiarization of the whole data set for each question; the identification of statements into codes of meaning; rank ordering of codes; the analysis of codes into superordinate themes; a review of statements, codes and themes; and report writing (Braun and Clark 2006).

### Presenter Focus Group

A focus group was held with the three presenters and the support services manager after TRT ended. Responses were recorded verbatim by the principle researcher. Field notes were also taken by the co-researcher as back up and a check of accuracy. Analysis involved the same quasi-qualitative approach used with participant interviews. Questions included: what presenters thought of TRT; what adolescents learned from the program; which parts of TRT worked better and what did presenters learn as a consequence of training in and delivery of TRT? Inter-rater reliability of interview and focus group data was conducted independently using the same procedure of analysis, by the principle researcher and a social science research assistant, experienced in quasi-qualitative analysis and is reported as Cohen’s kappa, *k.*

### Analysis

An Omnibus Multivariate analysis (MANOVA) was used to assess between group differences in TRT and waitlist SUDs, behavior and standardized measures. T-tests were used to assess pre and post-test differences within TRT and waitlist groups. The amount of ‘clinically’ significant change for individual adolescents was calculated by assessing the difference between pre and post-test SUDs for individual trauma targets as well as comparing differences in collated SUDs for all reported traumatic events for each adolescent. MANOVA was used to assess the influence of age and phase of TRT delivery (1, 2 and 3) on the outcomes measured. Because of the small sample size and skewed female population, gender was not analyzed as a moderating factor. Effect sizes (Cohen’s *d*) were calculated to determine the power of TRT in reducing participant subjective disturbance, PTSD, depression, dissociation, traumatic grief and mental health difficulties reported by staff. Effect size is reported as small (0.1–0.3), medium (0.4–0.6) and large effect (0.7–1.00). As attrition occurred prior to pre-intervention assessment, it was not possible to conduct an intention to treat analysis.

## Results

### Program Fidelity

As planned, all sessions were co-led by program workers and took place twice weekly. Sessions lasted 40 min on average keeping the duration within participants’ concentration spans. On average, three activities were covered per session. Three individual follow-up sessions were held with three individual participants because of missing one TRT session each. All missed their session because of a case review meeting. Adaptations to delivery involved utilizing smaller groups, and positive behavior management strategies because of challenging off-task behavior. Behavior problems were reported with one male and one female because of relationship split that spilled into the group and another male who did not view his difficulties as trauma based. Sessions 2 and 6 from phase two were randomly selected for inter-rater reliability analysis (*k* = .93).

#### Protocol Adherence

High levels of program protocols were maintained in the four sessions (94%). This was due to presenters following the order of activities and reading scripts in sessions. Adaptations to delivery occurred when a participant was struggling to understand a concept, unsure of the task, or concentration was waning towards the end of sessions. Presenters utilized a range of strategies to help adolescents comprehend and engage with tasks. For example, when an adolescent was unsure of what a bad dream was, examples were given of types of nightmare; the purpose of the activity was repeated; more time was spent on discussing and asking questions; and reassurance was given in not needing to draw the dream. Towards the end of sessions, presenters helped adolescents maintain focus by using affirmation statements, encouragement, and letting participants know there was only a short time left till the end of the session.

#### Presenter Communication

Presenters delivered TRT in calm warm tones throughout (100%), body language was open in nature (arms open; 92%), oriented towards participants (97%), and with high levels of eye contact (93%). While one of the presenters sat facing adolescents at a small Table (100%), the other would stand and write adolescent responses onto a flip chart (23%). As per protocols, sessions were recapped at the beginning for prompting memory of content, strategies learned, and the application of strategies. Questions were used to facilitate recall, e.g. “So what was it we covered last session?” “Do you remember ….”? All activities were introduced by stating the purpose of the activity and then checking for understanding.

Reception of adolescent response was high throughout the sessions (93%). Reception involved affirmation statements, summarizing what had been said, asking questions for clarification, and asking questions to elicit more details. Presenters frequently checked for student understanding, e.g. “So does that make sense? What do you think?” Presenters also checked whether adolescents had applied any of the strategies, reinforced the use of practice, and offered support. Presenters normalized different responses to activities and gave reassurance when adolescents were uncertain, e.g. “well done to take another turn.” Presenters took part in activities and modelled (i) the use of strategies and (ii) sharing thoughts and feelings. Presenters managed turn-taking by ensuring all adolescents got a turn, e.g., “so x, how did you change your dream?” In short, although high levels of TRT fidelity was achieved, adolescents needed a range of communicative strategies that enabled them to remain on task, take turns, recall learnings, understand what was being asked of them, and reflect on the strategies they used.

### SUDs

At pre-test, TRT and waitlist SUDs were not statistically different *F*(1,16) = 0.109, *p* = .746 (See Table 1). A difference, however, was found between the two groups at post-test with TRT reporting significantly lower levels of subjective distress, *F*(1,16) = 4.560, *p* < 0.50. A significant difference was found for TRT between pre and post-test (*t* = 5.034, *p* < .01) with a mean SUDs score reduction, *m* = 52.60 (*SD* = 17.34) to *m* = 26.40 (*SD* = 19.55). In contrast, no difference was found between pre and post-test SUDs in the waitlist (*t* = 2.166, *p* = .73). A slight reduction in waitlist mean scores occurred indicating a positive trend, *m* = 56.28 (*SD* = 28.83) to *m* = 46.14 (*SD* = 17.52). TRT achieved a large effect size, *d* = 1.10 for SUDs. No statistically significant SUDs results were found for the moderating factors of age and phase of TRT delivery.

**Table 1:** Comparison between TRT and waitlist (WL) - means, standard deviation, significance and effect size

Measure	Condition	Means (*SD*)		Significance	Effect size
		Pre	Post		
SUDS	TRT	52.60 (17.34)	26.40 (19.55)	*F*(1,16) = 4.560, *p* < 0.50*	*d* = 1.10
	WL	56.28 (28.83)	46.14 (17.52)		
PTSD	TRT	22.20 (13.68)	19.40 (11.51)	*F*(1,16) = 0.596, *p* = 452	*d* = 0.36
	WL	12.42 (16.51)	13.85 (18.22)		
Depression	TRT	26.10 (17.97)	24.20 (21.82)	*F*(1,16) = 0.193, *p* = .669	*d* = 0.35
	WL	15.29 (13.52)	30.00 (8.54)		
Dissociation	TRT	2.14 (1.47)	1.44 (1.22)	*F*(1,16) = 0.097, *p* = .761	*d* = 0.15
	WL	1.50 (1.68)	1.73 (2.35)		
T. Grief**	TRT	44.90 (19.47)	42.00 (16.03)	*F*(1,16) = 0.037, *p* = .851	*d* = 0.09
	WL	44 .00 (26.64)	43.86 (24.16)		
Mental Health	TRT	23.00 (3.23)	23.60 (3.44)	*F*(1,16) = 1.987, *p* = .180	*d* = 0.36
	WL	20.71 (4.99)	20.05 (5.43)		

*Significant result

**T Grief = Traumatic grief

In contrast to the waitlist, 4 TRT adolescents reported large reductions in their average SUDs for listed traumatic events: 31 to 7; 46 to 3; 65 to 10 and 34 to 17. Double the number of TRT adolescents reported individual trauma events rated as zero post-test (*n* = 20) compared to the waitlist (*n* = 10). Indications are TRT participants may have experienced clinical reductions in SUDs compared to the waitlist. This finding needs further research.

### Standardized Measures

Eleven adolescents fulfilled the criteria for the likelihood of PTSD (65%) and clinical depression (65%). Only three reported clinical levels indicative of dissociation (18%). No significant different was found at pre-test for PTSD, *F*(1,16) = 1.777, *p* = .202: depression, *F*(1,16) = 0.30, *p* = .866; dissociation, *F*(1,16) = 0.397, *p* = .541; and wider mental health difficulties, *F*(1,16) = 2288, *p* = .159. Following TRT, there was a trend in reduced symptoms for PTSD, depression, and traumatic grief compared to an increase in symptom levels for the waitlist. None of these results, however, were statistically significant. There was a high correlation between adolescent and staff reports of mental health difficulties at pre (0.94, *p* < .05) and post-test (0.618, *p* < .05) that supports adolescent self-reports.

### Behavior Monitoring

Behavior monitoring suggests adolescents in TRT and waitlist reduced in aggressive behavior (*t* = 4.589, *p* < .01 versus *t* = 6.61, *p* = .05) and upset (*t* = 3.000, *p* < .05 versus *t* = 1.4286, *p* < .05) pre to post-test, however, TRT showed greater trends towards change in aggression (*m* = 15.50 to *m* = 9.50 TRT versus *m* = 12.86 to *m* = 8.14 waitlist) and upset (*m* = 3.88 TRT to *m* = 1.50 versus *m* = 2.00 to *m* = 1.43 waitlist). Further, two TRT participants made substantial reductions in aggression (10 to 4 and 8 to 1 events respectively). This individual adolescent change was not apparent in the waitlist. Clinically, the most upsets (*n* = 10) for any single participant reduced to (*n* = 4) in TRT compared with *n* = 7 to *n* = 3 in the waitlist.

### Adolescent Interviews

**A**dolescent responses were short and indicated a wide range of perceived gains from TRT involvement (see Table 2). Adolescents were able to specify what aspects of TRT was most helpful, e.g. emotional regulation strategies, talking about experience, and dealing with intrusive thoughts, as well as what could be changed for the future (e.g. more individualized support). Nine adolescents reported TRT was ‘alright’ or ‘good’, the other reported that TRT “worked for others”. This latter adolescent commented “at the start I didn’t understand why I needed to tighten up and loosen muscles, but then realized at the end”. Six adolescents described the program as a “safe space.” Six adolescents also reported they didn’t like “groups.” Two found it “difficult to get pictures in the head” and one each found breathing; drawing; safe place and “another pupil jumping around” a challenge.

**Table 2:** Adolescent compared to worker perceptions

Issue	Adolescent perceptions	Worker perceptions
What liked / worked	Relaxing (*n* = 7); All activities (*n* = 6); Safe place (*n* = 4); Drawing (*n* = 2); Tapping (*n* = 1) Smelling (*n* = 1) Bad picture to good picture (*n* = 1) Being in a group (*n* = 1) Talking about things (*n* = 1) Comparing feelings then and now (*n* = 1)	Valuable contributions from adolescents (*n* = 27) Individual and group activities (*n* = 12) Imagery, graded exposure, fear thermometer, safe place, fun (*n* = 5) Emphasize purpose of the activity (*n* = 4) Visual materials to aid imagination (*n* = 4) Small groups & short sessions (*n* = 3)
What adolescents learned	Talking about feelings (*n* = 2) How to cope (*n* = 2) If annoyed, breathe and think about something else (*n* = 2) How to deal with difficult images, to keep them in the past (*n* = 2) How to put thoughts to the side (*n* = 2) Hear different points of view (*n* = 1) Beneficial to talk/not talk about (*n* = 1)	Normalization through shared experience (*n* = 9); Increased sense of control (*n* = 8); Re-visit learning in units (*n* = 7); Better understanding of trauma and symptoms (*n* = 6); Symptoms reduced (*n* = 4); Range of tools to apply in life (*n* = 4)
What workers learned	N/A	Extent of trauma (*n* = 10); Recognizing trauma events and symptoms including in reports (*n* = 9); Trauma lens report writing (*n* = 6); Trauma recovery strategies (*n* = 4); Helping agencies recognize trauma (*n* = 4); Revisiting learning for adolescents (*n* = 4); Cautious re asking about trauma (*n* = 3) Embed TRT into practice (*n* = 3); Trauma not recognized or met (*n* = 3); Change is not linear (*n* = 1)
Challenges	Not like groups (*n* = 6) Breathing, drawing and safe place (*n* = 3) Visual imagery (*n* = 2) Other adolescents’ behavior (*n* = 1)	Adolescent behavior (*n* = 17); Limited verbal contributions (*n* = 11); Liaison with care staff (*n* = 9); Uncertainty of adolescent response (*n* = 8); Need for follow-up to apply skills (*n* = 6); TRT delivery needed adapted (*n* = 5) Adolescents could respond to different activities on different days (*n* = 4)
Future directions	One to one TRT (*n* = 3) Individual work after group work (*n* = 1) More sessions (*n* = 1) Others need to open up more (*n* = 1) Not so much visualization (*n* = 1)	Liaising with care staff essential (*n* = 14); Encourage peer support (*n* = 10) Fun activites; visual aids and attractive workbook (*n* = 7) Selection and grouping important (*n* = 3) Shorter and more frequent sessions (*n* = 3)

Adolescents reported a range of activities they liked best: calming and relaxing (*n* = 7); all activities (*n* = 6); safe place (*n* = 4); drawing (*n* = 2); and one occurrence for tapping; smelling; “turning a bad picture into a good picture”; “being part of a group”; “talking about things”; and “comparing difference in feelings last time and now.” Adolescents reported on a range of what they learned: “talking about feelings” (*n* = 2); “how to cope with things” (*n* = 2); “if get annoyed, breathe and think about something else (*n* = 2)”; “how to deal with difficult images, to keep them in the past (*n* = 2)”; “to be able to put my thoughts to the side” (*n* = 2); “what’s beneficial to talk and not talk about”; and “to hear different points of view.”

In terms of rating the application of strategies beyond the group three adolescents scored ten, two scored eight, two scored six; and one scored five. One said, "Used safe place a couple of weeks ago when I thinking about something, made me forget what I was thinking about” and another “used it for sleep.” Another was “at least going to give them a try” and one “wasn’t sure”. For the future, three suggested to delivering TRT “one to one.” Other ideas included “individual work after group work”; “making TRT longer”; “others need to open up more”; and “don’t make it so repetitive (visualization techniques).”

### Focus Group Responses

Staff perceived adolescents as benefitting from TRT but emphasized the need to individualize TRT to fit with how adolescents respond in a group (see Table 2). Workers also reported a range of benefits for themselves including learning about the nature of trauma and how to deliver a trauma-specific program. Inter-rater reliability for analysis of adolescent interview and focus group data was *k* = .93 and *k* = .91 respectively.

Analysis of presenter responses identified ten codes from 95 statements. The rank order of codes was as follows: valuable contributions received from adolescents (*n* = 27); challenging behavior at times (*n* = 17); some limited verbal contributions (*n* = 11); encouraged support by peers (*n* = 10); liaison with care staff was essential (*n* = 9); uncertainty of adolescent response for presenters (*n* = 8); adolescents needed follow-up to apply skills (*n* = 6); TRT delivery needed adapted (*n* = 5) and it difficulties accessing photocopying facilities (*n* = 5). The above codes are summarized into the theme of ‘*Challenges of TRT delivery’.*

In terms of what adolescents learned, six codes were identified from 38 statements: normalization through shared experience (*n* = 9); increased sense of control (*n* = 8); chance to re-visit learning in real-life (*n* = 7); a better understanding of trauma events and symptoms (*n* = 6); reduced symptoms (*n* = 4); and a range of tools to apply in real-life (*n* = 4). The above codes are summarized into the theme of *‘Normalizing and coping’.*

Presenters perceived the following as working best in TRT: individual and group activities (*n* = 12). The activities of imagery, graded exposure, fear thermometer, and safe place as well as time for fun (*n* = 5) were reported as helpful. The importance of emphasizing the purpose of the activity (*n* = 4) and small groups enabled more focused interactions (*n* = 3). There was recognition that different adolescents responded to different activities (*n* = 2) and that the same adolescent could respond to different activities on different days (*n* = 2). The theme was *‘variety of structure and activity’*.

Presenters reported a wide range of learning for themselves. Fourteen codes were identified from 73 statements. These were as follows: The importance of liaising with care staff (*n* = 14); discovering the extent of trauma in adolescents (*n* = 10); recognizing trauma events and symptoms in adolescents’ written reports (*n* = 9); building in what adolescents respond to, e.g. fun; visual aids and attractive workbook (*n* = 7); writing reports from a trauma lens (*n* = 6); increased range of trauma recovery strategies (*n* = 4); helping other agencies recognize trauma (*n* = 4); needing to revisit learning for adolescents (*n* = 4); the importance of selection and grouping (*n* = 3); being cautious before asking about traumatic experiences (*n* = 3); how to embed TRT into practice and to keep learning (*n* = 3); increasingly feeling the negative impact of recognizing adolescents not being placed appropriately (trauma needs not recognized or met, *n* = 3); need to repeatedly emphasize the purpose of program and activities (*n* = 2) and accept that change is not linear (*n* = 1). *‘Trauma and program awareness, knowledge and skill development.’* was the theme.

## Discussion

The current study sought to stimulate discussion in the delivery of trauma-specific programs to high risk adolescents securely accommodated in Scotland. A secure facility was able to deliver a group-based trauma specific program to adolescents who had experienced multiple and cumulative abuse and trauma, and collaborate in conducting research including randomization of participants to conditions and qualitative interviews. Trauma history interviewing including SUDs, helped the secure facility identify specific traumatic events and related levels of internal distress for adolescents. This was pioneering work for the Scottish context where behavior change programs and the lack of rigorous evaluation is the norm (Barron and Mitchell 2016). Findings at this stage, however, are tentative. Reduced SUDs and trends towards less crisis situations, posttraumatic stress, depression and dissociation, suggest further development and evaluation of TRT would be helpful before recommendation for use.

Post-program SUDs, interviews, and presenter focus group responses all suggest that TRT can help high-risk adolescents begin to understand how normal the trauma reaction is as a response to adverse life events. Lowered SUDs suggests adolescents were beginning to reappraise traumatic events in their lives (Ronan et al. 2006). For example, adolescents reported that they discovered that they are not alone in what they were feeling and most were able to identify specific coping strategies learned and applied in the wider secure facility context. This was supported by presenter comments. More research, however, is needed into the relationship between reduced SUDs and program impact.

Despite the challenging nature of delivering a trauma-specific program in a secure facility, the current study identified that a high level of program fidelity is possible. Analysis of video material lead to the discovery that adaptation to the delivery of the porgram was less about changing program protocols and more related to the nature of presenter-adolescent communicative interactions. These included: (i) high levels of positive behavior management strategies to keep adolescents on task; (ii) pictures to support visualization for adolescents struggling with imagining; (iii) frequent reminders of the purpose of the activities; and (iv) involvement of care and education staff to encourage adolescents to use strategies beyond lessons. Presenters also emphasized the importance of participant selection for groups, keeping group sizes small and sessions of short duration. Although contested, a number of authors argue that such program adaptation can be an important part of program fidelity, as long as the changes are within program theoretical underpinnings (Backer 2001).

Interviews suggest adolescents were positive about TRT. Adolescents were to able identify a range of activities they enjoyed and provided useful ideas for future program adaptation. Some, however, were wary of receiving a trauma-specific intervention in a group. This may have been because of the risks associated with managing emotions as well as disclosure and confidentiality in front of adolescents they lived alongside (Kerig 2013). This raises questions about whether groups are the most effective context for delivering trauma-specific interventions for some adolescents in secure facilities. It may be that individual as well as group based trauma-specific programs need to be delivered. Mahoney et al. (2004) go further, to argue that because admission to secure can be traumatizing, therapeutic diversionary programs are more appropriate route to explore.

The current study further identified that program staff as well as adolescents report gains from delivering a trauma-specific program. These included re-conceptualizing adolescent challenging behavior as being underpinned by trauma, learning to utilize trauma-specific measures for screening and evaluation and discovering trauma-specific responses to intervening with traumatized adolescents. Significantly, being enabled to deliver a trauma-specific program led staff to want to “continue learning about adolescent traumatization and recovery”. These gains are not insignificant, as they begin to address gaps in current secure facilities in Scotland where the focus has mainly been on behavior rather than mental health (Barron and Mitchell 2016).

### Limitations

Because of the limited number of adolescents and secure facilities in Scotland, only a small sample was available. The sample of seventeen adolescents was well below the numbers needed for a program of medium power (*n* = 200) and as such a more positive program impact may not have been identified by this study. In addition, as adolescents were from the same facility, waitlist adolescents may have been indirectly exposed to information from the intervention, e.g., through changes in staff behavior or via peer interactions. This coupled with the small sample makes any conclusions potentially confounded by nonspecific factors such as the thoughtful attention of the presenters with the adolescents. The small and skewed nature of the sample towards girls did not permit any meaningful gender analysis to be conducted. The trauma history interview for eliciting and rating traumatic events has yet to be validated for program evaluation and was therefore experimental (Greenwald 2005). As measures were based on small clusters of traumas, they may not have been sufficiently sensitive to assess program change for adolescents’ experiencing cumulative trauma. The measure of traumatic grief may not have captured the symptoms of the emerging concept of multidimensional grief (Kaplow et al. 2013). The counting of interview and focus group statements and ranking of codes does not identify the significance of statements, simply the frequency. A single statement could therefore be more meaningful than a cluster. Program fidelity was limited as it was conducted with a small number of sessions. As program adaptation and fidelity refinements were made in the context of a randomized design, questions are raised regarding participants receiving the same type and level of treatment. The study was conducted in the complex environment of a traumatized population in a secure facility. Such an environment includes a rapid pace of change, heavy workloads for staff, high emotional demands and the unpredictability for staff and participants regarding placement moves (Liou 1995). These are all context factors that potentially impact program efficacy. Finally, adolescents heightened survival responses while in the facility may have been a factor in the effectiveness of TRT (Kerig 2013).

## Conclusions

The current study is an important addition to a new field of research in Scotland and adds to research globally in its infancy. The current study evidenced that it is possible to deliver a group-based trauma-specific program into a secure facility in the Scottish context. The program led to significant reductions in subjective disturbance for adolescents compared to those on the waitlist. Trends in reduced posttraumatic stress, depression and traumatic grief warrant further exploration. Likewise the trend in reduced behavioral incidents is promising given most adolescents are placed in secure facilities because of their behavioral difficulties. The study also highlights the need for program adaptation in a secure setting because of adolescent difficulties with motivation, concentration, comprehension of the purpose of activities and challenging behavior. Adaptations included the use of small groups, shorter and more frequent sessions, visual materials to aid imagination and positive behavior management to keep adolescents on task. Involvement of care staff was reported as essential in enabling adolescents to apply new coping skills beyond lessons. With these adaptations it appears program workers can achieve a surprisingly high level of fidelity. Finally, program workers reported gains the development of their conceptualization of adolescent trauma and trauma recovery as well as skills program delivery.

### Recommendations for Practitioners

The current study, highlights the potential to refine TRT for secure facilities in Scotland. The adaptability of TRT to the needs of adolescents and skills of the trainers are important topics for further development and research. It is recommended secure facilities should continue to explore the implementation and evaluation of trauma-specific approaches. Appropriately trained program staff are well placed to administer trauma-specific screening and evaluation measures. Program staff will need further training in the delivery and development of novel trauma-specific programs and trauma history interviewing. Care staff will need training in trauma-sensitive milieu in order to support trauma-specific interventions to help adolescents generalize their learning. Such training will need revisited, developed over time and supported by supervision (Sommer 2008). The videoing of program delivery provides one way of facilitating and reviewing program adherence for program workers. Finally, in addition to PTSD programs may be needed to address adolescent depression, dissociation, traumatic grief and wider mental health concerns.

### Recommendations for Research

Research into trauma-specific interventions is urgently needed for adolescents placed in secure facilities. Valid and reliable evaluation measures need to be developed for adolescents who have experienced cumulative domestic violence and who present with a range of developmental trauma symptoms. The concept and measures of multidimensional grief needs to be explored for adolescents in secure accommodation. Larger samples sizes are needed for empirical studies to compare gender as a moderating factor and aid generalization of findings. Future studies need to assess the validity and reliability of SUDs as a program evaluation measure. The effectiveness of trauma-specific group-based programs needs to be compared with individual trauma therapies. The identification of multiple needs and diagnosis of adolescents may assist future researchers consider the model of intervention. Finally, the potential for longer term gains need to be assessed.
